# Long‐Term Clinical Outcomes and Pain Assessment after Posterior Lumbar Interbody Fusion for Recurrent Lumbar Disc Herniation

**DOI:** 10.1111/os.12706

**Published:** 2020-06-03

**Authors:** Yalin Yang, Xu Yan, Wenhui Li, Weizong Sun, Kai Wang

**Affiliations:** ^1^ Department of Orthopedics The Second Hospital of Tianjin Medical University Tianjin China; ^2^ Department of Orthopedics Emergency Tianjin Hospital Tianjin China

**Keywords:** Clinical outcomes, Pain, Posterior lumbar interbody fusion, Quality of life, Recurrent lumbar disc herniation

## Abstract

**Objectives:**

The aim of this study was to investigate the long term effects of posterior lumbar interbody fusion (PLIF), applied after recurrent lumbar disc herniation (rLDH), on pain relief and clinical outcome improvement.

**Methods:**

The current study is a retrospective study. We observed 22 cases from 85 patients that had undergone PLIF during February 2003 to October 2012 and all patients were followed for at least 5 years. The average age of those patients were 53 years, among them there were eight men and 14 women. Plain radiography and dynamic plain films were obtained, pre‐operation, for every patient. Magnetic resonance imaging (MRI) or computed tomography (CT) was conducted to confirm the diagnosis of rLDH before the operation. All surgeries were performed from posterior approach by the same surgeon using PLIF. Quality of life (QOL) and clinical outcomes were assessed by Numerical Rating Scale (NRS), Japanese Orthopaedic Association (JOA) scoring system, and Oswestry Disability Index (ODI) before revision surgery and at 1 week, 3 months, 12 months, and 24 months postoperative. These were also examined every time they came back to the hospital for a review.

**Results:**

All patients were discharged and no serious comorbidities occurred. Three cases with wound infections and one case with dural laceration were cured and discharged. The end point of follow‐up was August 2018 and the mean follow‐up after revision surgery was 85 months. There were significant differences in NRS. It decreased from 7.32 ± 1.17 to 2.77 ± 1.31 (*P* < 0.05). The mean postoperative NRS score was 2.27 ± 1.48 (*P* < 0.05), 1.90 ± 1.51 (*P* < 0.05), and 2.36 ± 1.36 (*P* < 0.05) at 3, 12, and 24 months after surgery. There were no statistically significant differences (*P* > 0.05) in ODI scores. The average JOA score improved from 5.00 ± 1.08 to 8.18 ± 1.59 (*P* < 0.05) 1 week after revision surgery. RR was between 50% and 70%. Overall satisfaction rates were beyond 80%. Only one patient required subsequent lumber surgery during the follow‐up period.

**Conclusion:**

If surgical indications are mastered, undergoing PLIF after rLDH may induce efficient pain relief and major improvements in clinical outcome scores, as well as quality of life scores.

## Introduction

With the development of surgical techniques, an increasing number of people definitely choose surgery to treat the pain caused by lumbar spine. Compared with conservative therapies, surgical treatment essentially is economically favorable to relieve pain and enable early return to work^1^. However, if pain fails to improve or if new symptoms develop after spine surgery, patients are labeled with specifically failed back surgery syndrome (FBSS)^2^. As early as 1976, Crock first proposed “Failed spinal operation”, which was a failed spinal surgery, and divided it into four categories: postoperative symptoms were not relieved or aggravated; postoperative symptoms were re‐emphasized; postoperative lumbar spondylolisthesis progressed; and postoperative infection^3^. In the 1980s, Burton first proposed the concept of FBSS in his published article^4^. At present, FBSS is recognized at home and abroad as a type of syndrome of persistent or recurring lumbar back pain, with or without sciatica. The essence of its “failure” is that the results of lumbar surgery have not reached the expected value of the surgeon and the patient before surgery. The incidence rate is controversial, some scholars have reported that the incidence of FBSS is 10%–40% in all lumbar surgeries^5^, ^6^. A typical clinical feature of FBSS is chronic pain after surgery. There are many forms of pain, mainly divided into axial pain (pain mainly distributed in the waist) and root pain (pain distributed along the nerve root to the lower leg). In addition, pain can also involve the buttocks, hips, and thighs, which is the referred pain. Pain can also be divided into two types: mechanical pain (pain is aggravated when the trunk is loaded, bed rest can be relieved to varying degrees) and neuropathic pain (usually persistent pain along the nerve root distribution area and often combined with paresthesia). Because the symptoms of FBSS are usually localized and qualitatively blurred, they do not conform to the typical physiological distribution, which makes doctors confused.

Recurrent lumbar disc herniation(rLDH)is one of the most common etiologies for FBSS. Definitions of rLDH show great variations in different studies^7^, ^8^. The most common definition is disc herniation at the same level, regardless of ipsilateral or contralateral herniation, and recurrent back or leg pain after a definite pain‐free period lasting at least 6 months from initial surgery. Occurrence rates of re‐herniation range from 3% to 18%, according to retrospective studies^9^, ^10^, ^11^, ^12^. The most common clinical presentations of this disease are back pain and sciatica, which worsen after activity and are alleviated with rest. Recurrent lumbar disc herniation is associated with instability of the segment. The purpose of reoperation is to relieve pain and restore nerve function. Since the initial surgery changed the original normal anatomy, the scar tissue was severely adhered to the nerve, and the incision must be extended during the reoperation to enlarge the bone window. Although there are many research on the treatment of rLDH, treatment of rLDH is not standardized. Surgery is only one of the commonly used methods. Simple decompression does not involve fusion, and reoperation may further aggravate segmental instability, therefore, it is necessary to use a safe and effective method to treat rLDH.

Posterior lumbar interbody fusion (PLIF) is performed by retracting the posterior lamina and retracting the dural sac and nerve roots, then removing the intervertebral disc, and finally placing the graft or cage into the intervertebral space. PLIF is useful in managing degenerative disc disease, severe instability, spondylolisthesis, deformity, and pseudarthrosis and this procedure has obvious advantages such as laminectomy, full exposure, and complete decompression. The nucleus pulposus tissue can be completely removed during surgery, and the protrusion can be eliminated. The implantation of the interbody fusion cage can open the intervertebral space, restore and maintain the intervertebral height, restore the sagittal physiological sequence and curvature of the lumbar vertebrae, and eliminate the intractable lower back pain. However, PLIF has a high complication rate (dural tear, 5.4% to 10%; neurologic injury, 9% to 16%)^13^. In previous studies^14^, ^15^, ^16^. researchers were more concerned with treatment methods, proposing satisfied short‐term outcomes. However, few studies have focused on long‐term quality of life and clinical outcomes.

Based on those studies, the current study followed a series of patients with rLDH that had undergone PLIF for a long time. The purpose of this study is to explore the following questions: (i) Security: for patients with recurrence after discectomy, does this approach increase the risk of recurrence and will it affect the patients' life if combined with postoperative complications?; (ii) Effectiveness: PLIF can improve the stability of the spine, but at the same time reduce the mobility of the segment. For patients with rLDH, can this procedure significantly improve their symptoms and achieve good results?; (iii) Durability: Compared with short‐term satisfaction using other methods, can pain can be effectively relieved and can clinical outcomes be improved in the long‐term?

## Materials and Methods

This is a retrospective study of 22 selected cases from 85 patients with rLDH. Inclusion criteria included the following PICOS principle: (i) Participant: Patients who had a definite pain‐free period lasting at least 6 months after initial surgery and failed with conservative management for at least 8 weeks and the symptomatic recurrent herniation happened at the same level; (ii) Intervention: underwent PLIF between February 2003 to October 2012; (iii) Comparison: at least 5 years of self‐control outcome assessment; (iv) Outcome: pain assessment and clinical outcomes tests on a regular basis; and (v) Study Design: a retrospective study.

### *Patients*

Of these patients, there were eight men and 14 women, with an average age of 53 years (range, 34 to 68 years). Duration of the symptom ranged from 2 to 48 months (average, 13 months). Levels of re‐herniation were six participants in level L3‐L4, 19 participants in L4‐L5, and seven participants in L5‐S1, including multiple segments recurrence. The number of left and right herniation was 12 and 10, slightly different from previous studies. In those patients, four cases were combined with segmental instability. The mean follow‐up after revision surgery was 85 months (range, 62 to 182 months) (Table 1).

**Table 1:** Demographic and clinical characteristics of 22 patients with rLDH

Patient	Age (yr) /Gender	Symptoms and signs(duration)	Level of herniation	Follow‐up, (mos)	Pre/Post ODI (%)	Pre/Post NRS	RR^*1*^ (%)
1	62/F	Leg numbness, urinary retention (18mos)	L3‐4, L4‐5	66	52/13	6/2	44.4
2	44/F	Back pain and sciatica (3 mos)	L5‐S1	78	85/24	9/4	50.0
3	46/F	Lower–limb weakness and thigh tinging (5 mos)	L5‐S1	92	65/35	7/3	33.3
4	50/F	Motor Weakness in foot dorsiflexion and sciatica(6mos)	L4‐5, L5‐S1	65	68/14	7/3	50.0
5	65/M	Back pain and sensory deficit (24mos)	L4‐5	84	78/16	9/3	57.1
6	48/F	Back pain and sciatica (6 mos)	L3‐4, L4‐5	70	80/26	9/4	60.0
7	51/F	Lower–limb weakness and sciatica (4 mos)	L4‐5, L5‐S1	66	80/26	7/5	25.0
8	46/M	Sciatica and lower limb weakness (12mos)	L4‐5	78	62/4	7/1	100.0
9	64/F	Numbness, weakness and urinary incontinence (48mos)	L4‐5	62	73/8	8/2	62.5
10	52/F	Back pain and sensory deficit (24mos)	L4‐5	88	75/19	9/3	66.7
11	59/M	Sensory deficit, lower limb weakness (4mos)	L4‐5, L5‐S1	69	64/18	8/3	71.4
12	51/F	Back and leg pain (16 mos)	L4‐5	72	57/9	7/2	50.0
13	61/M	Numbness, tingling and lower limb weakness (14mos)	L3‐4, L4‐5	83	68/12	8/3	60.0
14	50/F	Back pain and sciatica (2 mos)	L4‐5	92	57/6	7/1	75.0
15	47/F	Lower–limb motor Weakness and sensory deficit (7 mos)	L3‐4, L4‐5	80	45/14	5/2	50.0
16	62/M	Back and leg pain (36 mos)	L4‐5	70	74/54	8/6	14.3
17	52/M	Weakness in flexion of the great toe and sciatica (3 mos)	L4‐5, L5‐S1	66	50/9	5/1	57.1
18	57/F	Back and leg pain (28mos)	L4‐5	88	68/10	7/2	75.0
19	34/M	Back pain, sciatica and thigh tinging (3 mos)	L4‐5	182	56/10	6/1	80.0
20	46/F	Lower limb weakness, pain and numbness (6mos)	L4‐5, L5‐S1	126	57/14	7/3	50.0
21	52/F	Sensory deficit, lower–limb weakness and pain (6 mos)	L3‐4, L4‐5	98	65/16	8/3	50.0
22	68/M	Intermittent claudication and sensory deficit (12 mos)	L3‐4	100	52/26	7/4	44.4

F, female; M, male; mos, months; NRS, numerical rating scale; ODI, Oswestry Disability Index; Pre., preoperative; Post, postoperative; RR, recovery rate.

^*1*^RR = 100% means complete healing; RR>60% means remarkable effective; RR between 25% to 60% means effective and <25% means ineffective.

**Table 2:** Japanese Orthopedic Association Scores (JOA score)

I. Subjective symptoms (9 points)			
A. Low‐back pain			
(a) None		3	
(b) Occasional mild pain		2	
(c) Frequent mild or occasional severe pain		1	
(d) Frequent or continuous severe pain		0	
B. Leg pain and/or tingling			
(a) None		3	
(b) Occasional slight symptoms		2	
(c) Frequent slight or occasional severe symptoms		1	
(d) Frequent or continuous severe symptoms		0	
C. Gait			
(a) Normal		3	
(b) Able to walk farther than 500 m, although it results in pain, tingling, and/or muscle weakness		2	
(c) Unable to walk farther than 500 m because of leg pain, tingling, and/or muscle weakness		1	
(d) Unable to walk farther than 100 m because of leg pain, tingling, and/or muscle weakness		0	
II. Clinical signs (6 points)			
A. Straight‐leg raising test (including tight hamstrings)			
(a) Normal		2	
(b) 30°‐70°		1	
(c)<30°		0	
B. Sensory disturbance			
(a) None		2	
(b) Slight disturbance (not subjective)		1	
(c) Marked disturbance		0	
C. Motor disturbance (manual muscle testing)			
(a) Normal (grade 5)		2	
(b) Slight weakness (grade 4)		1	
(c) Marked weakness (grade 3–0)		0	
III. Restriction of activities of daily living (14 points)			
Activities of daily living	Severe	Moderate	None
Turning over while lying	0	1	2
Standing	0	1	2
Washing	0	1	2
Leaning forward	0	1	2
Sitting (1 hr)	0	1	2
Lifting or holding	0	1	2
Walking	0	1	2
IV. Urinary bladder function (−6 points) (incontinence, urinary retention)			
(a) Normal		0	
(b) Mild dysuria		−3	
(c) Severe dysuria		−6	

### *Radiographic Measurement*

**Figure 1:**
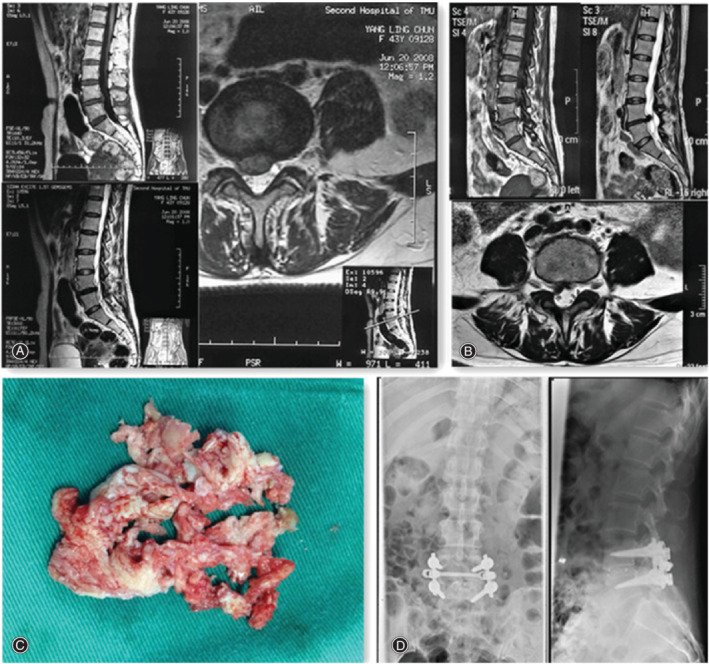
Illustration case: A 46‐year‐old woman received PLIF as revision surgery. (A) Sagittal T1‐weighted and Axial T2‐weighted MRI demonstrating intervertebral disc herniation at L4‐L5 level before the initial surgery. (B) Sagittal and Transverse T2‐weighted MRI indicating recurrence in the same segment after 4 years. (C) Intervertebral disc tissue removed during the second operation. (D) Anteroposterior and lateral view of X‐ray film after PLIF.

**Figure 2:**
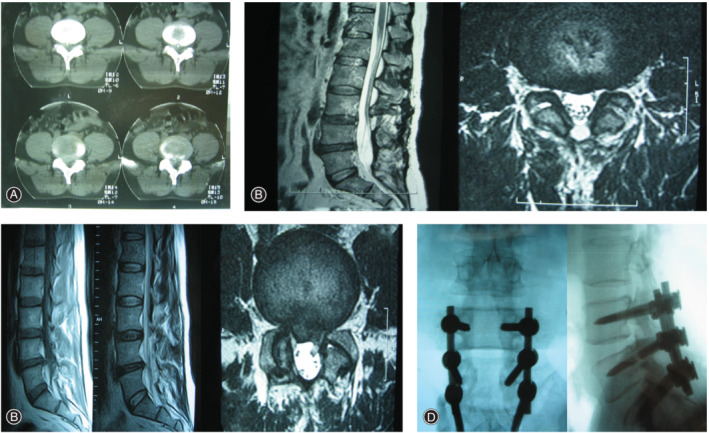
Illustration case: A 52‐year‐old male received PLIF as revision surgery. (A) CT transverse position demonstrating L4/L5, L5/S1 lumbar disc herniation with calcification and spinal stenosis. (B) Sagittal and Transverse MRI indicating L4 and L5 total laminectomy and decompression for the initial surgery. (C) The second preoperative MRI sagittal and transverse‐section showed recurrence of lumbar disc herniation, showing the image of old surgical scars. (D) Anteroposterior and lateral view of X‐ray film after PLIF (L4/L5/S1).

**Figure 3:**
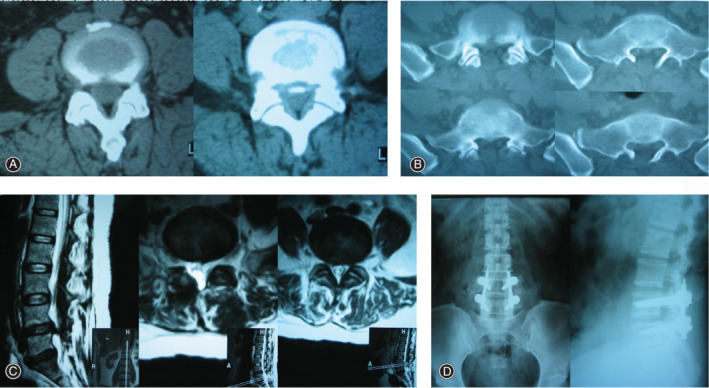
Illustration case: A 46‐year‐old woman received PLIF as revision surgery. (A) CT transverse position demonstrating L4/L5 lumbar disc herniation with calcification and spinal stenosis. (B) CT transverse position shows L4, L5 total laminectomy and decompression. (C) The second preoperative MRI sagittal and transverse‐section showed recurrence of lumbar disc herniation, showing the image of old surgical scars. (D) Anteroposterior and lateral view of X‐ray film after PLIF.

All patients with recurrent symptoms after primary surgery received plain radiographies to eliminate fractures or discitis. Magnetic resonance imaging (MRI) or computed tomography (CT) was then conducted to confirm the diagnosis of rLDH, evaluating the degree of nerve or spinal cord compression. Finally, dynamic plain films were obtained to calculate range of motion (ROM) of the disc and assess segment instability (Figs 1, 2, 3). All patients underwent plain radiographies and dynamic plain films. Thirteen patients underwent CT (including one with CT three‐dimensional imaging), 17 patients underwent MRIs (including four patients with gadolinium‐enhanced MRI), and three patients accepted myelography with X‐rays. Serial imaging was obtained at 3, 6, and 12 months after revision surgery of every patient. If there was no discomfort after the operation, the patients were informed that they should review the plain X‐rays (anteroposterior and lateral views) every year or two. CTs or MRIs were taken at any time of recurrent symptoms.

### *Surgical Techniques*

Two cases suffered back surgeries twice while others underwent only one. The initial operation method included removal of pulpous nuclear in three patients, fenestration decompression in five patients, hemilaminectomy in eight patients, and total laminectomy in four patients. Two of them used internal fixation without fusion and a microdiscectomy was performed in two cases. In this series, two patients underwent total laminectomies as revision surgery, experiencing rLDH once again. The interval time between primary surgery and repeat operations was 34 months (range, 11 to 64 months).

All surgeries were performed from posterior approach by the same surgeon using PLIF. The technique was carried out as follows: (i) position: prone position; (ii) incision: the posterior median incision of the lumbar region centered on the herniated segment; (iii) exposure: revealing bilateral lamina and facet joints of the herniated segment; (iv) decompression range: removing the lower third of the lamina of the adjacent vertebral body, and half of the medial aspect of the facet joint, including excising the overlapping lower articular process and the lateral portion of the lamina; (v) excision of the ligamentum flavum, pulling the dura and nerve roots to one side, revealing the annulus fibrosus; (vi) use a small knife to cut the annulus fibrosus, curette to remove the nucleus pulposus tissue, and the endplate curette to scrape the endplate cartilage; (vii) intervertebral bone grafting and place interbody fusion cage; and (viii) pedicle screw fixation.

#### *Pain Assessment and Clinical Outcomes*

Changes in postoperative pain, quality of life, and outcome scores were evaluated using the Numerical Rating Scale (NRS)^17^, Japanese Orthopedic Association (JOA) scoring system^18^ (Table 2), and Oswestry Disability Index (ODI)^19^.

**Table 3:** Modified MacNab criteria

Excellent	Modified MacNab criteria
	No pain, no restriction of activity
Good	Occasional back or leg pain with enough severity to interfere with the patients ability to do their normal work or their capacity to enjoy themselves in their leisure hours
Fair	Improved functional capacity but handicapped by intermittent pain of enough severity to curtail or modify work or leisure activities
Poor	No or insufficient improvement to enable increase in activities; further operative intervention required

##### Numerical Rating Scale (NRS)

NRS definition: NRS allows a person to describe the intensity of his/her pain as a number usually ranging from 0 to 10, where “0“ means “no pain“ and “10“ means pain as “bad as it could be“.

##### Japanese Orthopedic Association (JOA) Scoring System

The JOA scoring system is a physician‐based outcome score that is mainly used to evaluate functional disorders in humans. The JOA total score is 29 points and the lowest is 0 points. The lower the score, the more obvious the dysfunction. Improvement index = post‐treatment score ‐ pre‐treatment score, post‐treatment score improvement rate = (post‐treatment score ‐ pre‐treatment score) / (29 ‐ pre‐treatment score) × 100%. By improving the index, it can reflect the improvement of lumbar function before and after treatment. The improvement rate can be used to understand the clinical treatment effect. The improvement rate may also correspond to the commonly used efficacy determination criteria: the improvement rate is 100% for the cure, the improvement rate is greater than 60% for the effect, 25%–60% is effective, and less than 25% is invalid.

##### Oswestry Disability Index (ODI)

Oswestry disability index (ODI) is a principal condition‐specific outcome measure used in the management of spinal disorders, and to assess patient progress in routine clinical practice. The ODI score system includes 10 sections: pain intensity, personal care, lifting, walking, sitting, standing, sleeping, sex life, social life, and traveling. For each section of six statements the total score is 5. Intervening statements are scored according to the list of options. If more than one box is marked in each section, take the highest score. If all 10 sections are completed the score is calculated as follows: total scored out of total possible score × 100. If one section is missed (or not applicable) the score is calculated: (total score/(5 × number of questions answered)) × 100%. Scores are as follows: 0%–20% is considered mild dysfunction, 21%–40% is moderate dysfunction, 41%–60% is severe dysfunction, and 61%–80% is considered as disability. For cases with score of 81%–100%, patients are either long‐term bedridden, or exaggerating the impact of pain on their life.

Patients completed the questionnaires before revision surgery and at 1 week, 3 months, 12 months, and 24 months postoperative. This study used the recovery rate (RR) of JOA and general scores of ODI and NRS as the evaluation index. The recovery rate was calculated as^20^:

RR = (postoperative JOA score ‐ preoperative JOA score) / (29‐preoperative score) × 100%.

Additionally, postoperative outcomes were divided into four‐grade on the basis of the modified MacNab criteria^21^ (Table 3). The overall satisfaction rate was combined with excellent and good results.

**Table 4:** Quality of life and clinical outcomes at time of follow‐up

Group		NRS (mean ± SEM)	ODI % (mean ± SEM)	RR(%)	Overall satisfaction rate(%)
Preoperative	1 week	7.32 ± 1.17	65.04 ± 10.99	—	—
Postoperative	3 months	2.77 ± 1.31*	17.41 ± 11.24	55.74 ± 18.72	86.4
	12 months	2.27 ± 1.48*	14.59 ± 8.08	69.14 ± 14.58	86.4
	24 months	1.90 ± 1.51*	13.36 ± 7.59	63.25 ± 13.78	90.9
		2.36 ± 1.36*	14.73 ± 7.85	58.23 ± 20.22	90.9
Last follow‐up		3.00 ± 1.45	16.27 ± 7.56	51.81 ± 17.56	86.4

ODI, Oswestry Disability Index; NRS, numerical rating scale; RR, recover rate; SEM, standard error of the mean.

* Statistically significant (*P* < 0.05).

### *Statistical Analysis*

Student's t‐test was used to compare data and parametric data are presented as mean ± standard deviation (SD). All data were analyzed using Statistical Package for the Social Sciences 16.0 (SPSS 16.0, IBM corporation), with *P* < 0.05 indicating statistical significance.

## Results

### *Patients' Follow‐Up and General Results*

All the patients recruited for this study signed an informed consent and were followed up by phone, e‐mail, or outpatient review. The starting time of the study was the time of the patient's operation, and the end time was the time of the end of the follow‐up. The mean follow‐up after revision surgery was 85 months (range, 62 to 182 months).

The average operation time was 168 minutes (rang: 122 to 231). Loss of blood varied from 380 to 600 mL (Average: 412 mL). The longest hospital stay was 21 days.

### *Pain Assessment*

In this series, most patients announced that both back pain and sciatic pain significantly and immediately improved after surgery. The straight‐leg raising test turned into normal conditions, gradually. Only three patients were not satisfied with the surgery, complaining that the outcome did not live up to their expectations, even if their symptoms were alleviated to varying degrees.

### *Clinical Outcomes*

**Figure 4:**
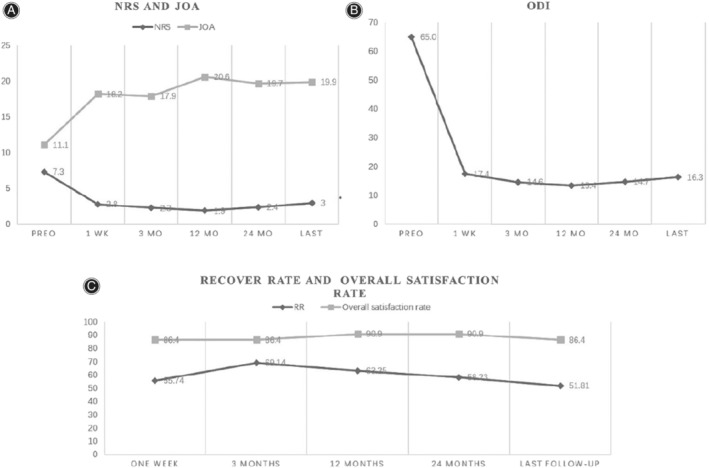
Clinical outcomes of patients: (A) Mean NRS and JOA scores over time. (B) Mean ODI scores over time. (C) The recovery rate and overall satisfaction rate of 22 patients.

Generally, the average ODI score improved from 65.04 ± 10.99 to 17.41 ± 11.24 (t = 18.27, *P* = 0.07) 1 week after the operation. At 3, 12, and 24 months post‐operation, it improved to 14.59 ± 8.08 (t = 20.91, *P* = 0.14),13.36 ± 7.59(t = 22.18, *P* = 0.11),14.73 ± 7.85(t = 21.22, *P* = 0.12) (Fig. 4). There were no statistically significant differences (*P* > 0.05) in ODI scores. However, there were significant differences in NRS and JOA scores (Fig. 4). The NRS decreased to 2.77 ± 1.31(t = 17.42, *P* = 0.01) 1 week after surgery and the mean postoperative NRS score was 2.27 ± 1.48(t = 17.38, *P* = 0.02), 1.90 ± 1.51(t = 19.56, *P* = 0.007), and 2.36 ± 1.36(t = 18.01, *P* = 0.02) at 3, 12, and 24 months after surgery (Table 4).

The mean recovery rate was 51.81 ± 17.56 (range, 25% to 100%). Overall satisfaction rate was 86.4% until the last follow‐up (Fig. 4).

### *Complications*

Postoperative complications included wound infections (three cases), which healed after the use of antibiotics and regular wound dressing. The patients' symptoms were completely relieved during the postoperative period, the body temperature was not high, the C‐reactive protein (CRP) and the erythrocyte sedimentation rate (ESR) were slightly higher. At 10 days after the operation, one patient developed a low‐toxicity infection with fat liquefaction in the wound. The sutures were taken out at 14 days post operation. After the dressing change, laser baking, and local debridement, the patients’ wound were healed. We considered the reasons may be local tissue blood flow disorders caused by excessive intraoperative stretching and improper use of electric knife, coupled with the poor nutritional status of the patient.

One patient with dural laceration and healed after continuous drainage and delayed extubation. The patient's symptoms were relieved post operation and back pain was significantly relieved. Considering a lot of clear liquid was drained post operation and body temperature was normal, we thought the patient may have ruptured the dura mater. In order to avoid dural rupture, the layers of the incision need to be sutured tightly and the paravertebral muscles should be closely aligned, which was conducive to the rapid healing of the muscles. Pay particular attention to the suture of the fascia lumbodorsalis and the needle pitch should be dense to prevent cerebrospinal fluid from entering the incision. When conducting extubation, the skin of the drainage tube mouth should be properly trimmed to make it a fresh wound and tightly sutured, which is beneficial to the closure of the drainage mouth at an early date. One patient required subsequent lumber surgery during the follow‐up period.

### *Difficulties in Revision*

There is no normal anatomical relationship at the time of initial surgery during revision surgery. Due to local soft tissue adhesion, revision is prone to damage important tissues such as blood vessels, nerves, and dural sacs, which significantly increase the difficulty and risk of exposure. Spinal cord drift and position variability after laminectomy during the first operation. Due to the formation of scar tissue around the dural sac, it isvery difficult to decompose adhesions. If improper operation is performed, dural sac injury or even catastrophic spinal cord injury is very likely to occur during the operation.

## Discussion

The development of diagnostic and surgical techniques makes it easier to accurately highlight intervertebral disc herniation. Patients pretend to take a more active approach to solve the problem of back pain. Compared with conservative management, surgical intervention is usually recommended, providing faster relief and improving symptoms rapidly^22^, ^23^. However, a series of problems come with surgery. One of the most common problems faced is the recurrence of symptoms caused by rLDH.

The most common clinical presentation of recurrence of symptoms includes back and radiating pain (in a distribution that correlated with herniation), debilitating symptoms (motor weakness, bilateral thigh and leg heaviness, or fatigue), and nerve root tension signs (positive straight leg raising test). These symptoms have caused severe suffering for patients, presenting a great challenge to surgeons.

As one of the most common complications of back surgery, there are still no good ways to avoid rLDH. No guidelines or significant comparative studies are available to assist surgeons in determining which approach would be most appropriate to treat rLDH^24^. There remains great controversy with surgical treatment. In a previous study^25^, one‐third of recurrent herniation cases were effectively managed with conservative therapy alone. They suggested that prolonged conservative management should be attempted. Moreover, a repeat discectomy is generally more difficult, due to scar tissue from the primary surgery and indistinct anatomical plans. There is an increased risk of complications, such as nerve root injury or dural tears^14^, ^24^, ^26^. Thus, whether surgery is still the first option for patients with rLDH is unknown.

In previous studies, short‐ and long‐term outcomes were satisfied^27^, ^28^. On the contrary, Vik *et al*. performed a retrospective study comparing the results of 39 patients that underwent reoperations with 124 patients operated on only once. Eight years after the operation for lumbar disc herniation, outcomes were significantly worse in re‐operated patients than in patients operated on only once^29^. Lebow *et al*. presented a prospective cohort study of 108 patients undergoing lumbar discectomy. Within 2 years of follow‐up, 25 patients experienced symptomatic or silent recurrence. Patient‐reported outcomes (LP‐VAS and ODI) were significantly worse after revision discectomies compared to primary discectomies^9^. This suggests that revision discectomies may be less successful at relieving symptoms associated with disc herniation than primary discectomies. This may be related, however, to different inclusion criteria, length of the follow‐ups, and other reasons.

Therefore, the current study explored the persistence of pain relief, quality of life, and long‐term clinical outcomes after PLIF for rLDH. To minimize the effects of preoperative factors on present results, patients were excluded due to the following: age beyond 70, smoking, diabetes, or alcohol use. There were no special restrictions on genderation, herniation type, and other variables.

After at least 5 years of follow‐ups, most of the recurrent cases were women. The gender distribution of patients (M:F = 8:14) was not observable, as with previous studies^30^, ^31^. This study excluded patients with a history of smoking, most of whom were male. Kim *et al*. argued that smoking can inhibit the annular healing process after discectomies and increase incidence of rLDH^8^. In addition, this study found that segments of herniation were mostly concentrated in the L4‐L5 level (19 patients, 86.4%). This may be related to the anatomy of the spine, which leads to great stress at the L4‐L5 level. Stress conditions for a long time result in degenerative changes in histopathology and biomechanics capability of lumbar intervertebral discs.

In the current study, all patients underwent PLIF, whether they combined segmental instability with lumbar spondylolisthesis or not. Although fusion may lead to good early clinical results, it does not seem to provide enough stability, according to longer follow‐up^32^. However, during present long‐term follow‐ups, the effectiveness of the operations was good. PLIF can preserve a solid fusion and recover a natural spinal anatomy, with limited excessive motion.

Of these patients, symptomatic improvement was not obvious in three patients. They still complained of back or leg pain and limited mobility. Four patients suffered intermittent attacks of moderate or severe back or leg pain, according to long‐term follow‐ups. They were relieved by analgesics or rest. One patient required subsequent lumber surgery.

Fortunately, all patients were discharged after recovery and no serious comorbidities, such as durotomies, never root injury, and massive hemorrhaging caused by vascular injuries, occurred. Wound infections occurred in three cases (13.6%), but finally healed with the use of antibiotics and regular wound dressing. One case of dural laceration (4.5%) was cured by conservative treatment. In order to reduce complications, the surgeon should be familiar with the anatomy of the operation area before surgery and read CT, MRI, angiography, and other imaging data in detail. With the help of 3D printed models, surgeons should familiarize themselves with the adjacent relationship of important anatomical structures, and fully consider the local anatomical changes caused by the initial operation. The reasonable surgical approach, resection method, and internal fixation reconstruction were selected, and the surgical plan was carefully formulated. Fully estimate the difficulty of revision surgery, formulate a plan to deal with various situations that may occur during surgery, and consult with relevant specialists when necessary, make full preparations, have sufficient blood, and have internal fixation tools. Do not rush into battle. During the operation, the normal tissues should be carefully exposed and distinguished to avoid damaging critical blood vessels, nerves, and organs. It is recommended to use a microscope during surgery to improve the resolution accuracy, and to use blunt and sharp separation techniques to free and remove tissue.

Intraoperative controlled blood pressure reduction, comprehensive use of vascular ligation, bipolar electrocoagulation, hemostatic material compression and other hemostatic methods to effectively control bleeding, according to the preoperative plan and orderly procedure.

Time of pain relief duration, which has not been examined in previous studies, is an important manifestation of a successful surgery. However, researchers have been more concerned with treatment methods, risk factors, or proposed satisfied short‐term outcomes^10^, ^33^, ^34^. Outcomes of revision surgery are varied. This may be due to the selection of the operation, time of follow‐up, and differences in inclusion criteria.

In the current study, pain was relieved or removed obviously after surgery. During follow‐ups, NRS scores decreased significantly (*P* < 0.05). Approximately 86% of the patients had good or excellent outcomes. Perhaps due to the limited number of patients, there were no statistically significant differences (*P* > 0.05) in ODI scores, although the mean ODI improved by more than 40%. The overall satisfaction rate was beyond 80.0%.

However, several limitations to the current study cannot be ignored. First, the initial operation was not performed by the single surgeon, perhaps leading to different degrees of difficulty in reoperations and influencing clinical results. Second, the design of this study did not set up a control group. Clinical outcomes were compared preoperatively and postoperatively, in which primary discectomies were not included.

### *Conclusion*

PLIF for rLDH can obviously improve symptoms for most patients. Pain can be alleviated for a long time. Quality of life and long‐term outcomes are desirable.

## References

[1] Hout WB , Den V , Peul WC , Koes BW , *et al* Prolonged conservative care versus early surgery in patients with sciatica from lumbar disc herniation: cost utility analysis alongside a randomised controlled trial. BMJ, 2008, 336: 1351–1354. 18502912 10.1136/bmj.39583.709074.BE PMC2427123

[2] Villavicencio AT , Nelson E L, Burneikiene S , et al. Surgical treatment strategies for the previously operated lumbar spine. Contemp Spine Surg, 2012, 13: 1–7.

[3] Crock HV . Observations on the management of failed spinal operations. J Bone Joint Surg Br, 1976, 58: 193–199. 932081 10.1302/0301-620X.58B2.932081

[4] Burton CV , Kirkaldy‐Willis WH , Yong‐Hing K , *et al* Causes of failure of surgery on the lumbar spine. Clin Orthop Relat Res, 1981, 157: 191–199. 7249453

[5] Voormolen J . In: Wilkinson HA, ed. The Failed Back Syndrome: Etiology and Therapy, Vol. 94, 2nd edn Berlin/Heidelberg/New York, 254 pages, DM 138.00 (hard cover), ISBN 3‐540‐97617‐5: Springer‐Verlag, 1992; 275–275.

[6] North RB , Kidd DH , Zahurak M , James CS , Long DM . Spinal cord stimulation for chronic, intractable pain: experience over two decades. Neurosurgery, 1993, 32: 384–394. 8455763 10.1227/00006123-199303000-00008

[7] Lee JK , Amorosa L , Cho SK , Weidenbaum M , Kim Y . Recurrent lumbar disk herniation. J Am Acad Orthop Surg, 2010, 18: 327–337. 20511438 10.5435/00124635-201006000-00005

[8] Kim KT , Park SW , Kim YB . Disc height and segmental motion as risk factors for recurrent lumbar disc herniation. Spine, 2009, 34: 2674–2678. 19910771 10.1097/BRS.0b013e3181b4aaac

[9] Lebow RL , Adogwa O , Parker SL , Sharma A , Cheng J , McGirt MJ . Asymptomatic same‐site recurrent disc herniation after lumbar discectomy: results of a prospective longitudinal study with 2‐year serial imaging. Spine (Phila Pa 1976), 2011, 36: 2147–2151. 21343849 10.1097/BRS.0b013e3182054595

[10] Papadopoulos EC , Girardi FP , Sandhu HS , *et al* Outcome of revision discectomies following recurrent lumbar disc herniation. Spine, 2006, 31: 1473–1476. 16741457 10.1097/01.brs.0000219872.43318.7a

[11] Heindel P , Tuchman A , Hsieh PC , *et al* Reoperation rates after single‐level lumbar discectomy. Spine (Phila Pa 1976), 2017, 42: 496–501. 10.1097/BRS.0000000000001855 27548580

[12] Parker SL , Mendenhall SK , Godil SS , *et al* Incidence of low Back pain after lumbar discectomy for herniated disc and its effect on patient‐reported outcomes. Clin Orthop Relat Res, 2015, 473: 1988–1999. 25694267 10.1007/s11999-015-4193-1 PMC4419014

[13] Dipaola CP , Molinari RW . Posterior lumbar interbody fusion. J Am Acad Orthop Surg, 2008, 16: 130–139. 18316711 10.5435/00124635-200803000-00004

[14] O'Donnell JA , Anderson JT , Haas AR , *et al* Treatment of recurrent lumbar disc herniation with or without fusion in workers' compensation subject. Spine, 2017, 42: E864–E870. 28700387 10.1097/BRS.0000000000002057

[15] Makino T , Kaito T , Fujiwara H , *et al* Risk factors for poor patient‐reported quality of life outcomes after posterior lumbar interbody fusion: an analysis of 2‐year follow‐up. Spine (Phila Pa 1976), 2017, 42: 1502–1510. 28248893 10.1097/BRS.0000000000002137

[16] Carragee EJ , Han MY , Suen PW , *et al* Clinical outcomes after lumbar discectomy for sciatica: the effects of fragment type and anular competence. J Bone Joint Surg Am, 2003, 85: 102–108. 12533579

[17] Farrar J , Young JJ , Lamoreaux L , *et al* Clinical importance of changes in chronic pain intensity measured on an 11‐point numerical pain rating scale. Pain, 2001, 94: 149–158. 11690728 10.1016/S0304-3959(01)00349-9

[18] Ohtori S , Ito T , Yamashita M , *et al* Evaluation of low Back pain using the Japanese Orthopaedic Association back pain evaluation questionnaire for lumbar spinal disease in a multicenter study: differences in scores based on age, sex, and type of disease. J Orthop Sci, 2010, 15: 86–91. 20151256 10.1007/s00776-009-1426-8

[19] Fairbank JC , Pynsent PB . The Oswestry disability index. Spine, 2000, 25: 2940–2953. 11074683 10.1097/00007632-200011150-00017

[20] Dai LY , Zhou Q , Yao WF , Shen L . Recurrent lumbar disc herniation after discectomy: outcome of repeat discectomy. Surg Neurol, 2005, 64: 226–231. 16099250 10.1016/j.surneu.2004.11.003

[21] Obenchain TG . Speculum lumbar extraforaminal microdiscectomy. Spine J, 2001, 1: 415–420. 14588298 10.1016/s1529-9430(01)00149-8

[22] Awad JN , Moskovich R , Awad JN , *et al* Lumbar disc herniations: surgical versus nonsurgical treatment. Clin Orthop Rel Res, 2006, 443: 183–197. 10.1097/01.blo.0000198724.54891.3a 16462442

[23] Gibson JN , Waddell G . Surgical interventions for lumbar disc prolapse: updated Cochrane review. Spine, 2007, 32: 1735–1747. 17632394 10.1097/BRS.0b013e3180bc2431

[24] Drazin D , Ugiliweneza B , Al‐Khouja L , *et al* Treatment of recurrent disc herniation: a systematic review. Cureus, 2016, 8: e622. 27382530 10.7759/cureus.622 PMC4922511

[25] Ambrossi GL , McGirt Matthew J,Sciubba Daniel M et al. Recurrent lumbar disc herniation after single‐level lumbar discectomy: incidence and health care cost analysis. Neurosurgery, 2009, 65: 574–578. 19687703 10.1227/01.NEU.0000350224.36213.F9

[26] Cinotti G , Roysam GS , Eisenstein SM , Postacchini F . Ipsilateral recurrent lumbar disc herniation. A prospective, controlled study. J Bone Joint Surg Br, 1998, 80: 825–832. 9768893 10.1302/0301-620x.80b5.8540

[27] Ambrossi GL , McGirt MJ , Sciubba DM , *et al* Recurrent lumbar disc herniation after single‐level lumbar discectomy: incidence and health care cost analysis. Neurosurgery, 2009, 65: 574–578; discussion 578. 19687703 10.1227/01.NEU.0000350224.36213.F9

[28] Yorimitsu E , Chiba K , Toyama Y , Hirabayashi K . Long‐term outcomes of standard discectomy for lumbar disc herniation: a follow‐up study of more than 10 years. Spine, 2001, 26: 652–657. 11246379 10.1097/00007632-200103150-00019

[29] Vik A , Zwart JA , Hulleberg G , *et al* Eight year outcome after surgery for lumbar disc herniation: a comparison of reoperated and not reoperated patients. Acta Neurochir, 2001, 143: 607–611. 11534678 10.1007/s007010170066

[30] Suk KS , Lee HM , Moon SH , Kim NH . Recurrent lumbar disc herniation: results of operative management. Spine, 2001, 26: 672–676. 11246384 10.1097/00007632-200103150-00024

[31] Kim KT , Lee DH , Cho DC , Sung JK , Kim YB . Preoperative risk factors for recurrent lumbar disc herniation in L5‐S1. J Spinal Disord Tech, 2015, 28: E571–E577. 25089673 10.1097/BSD.0000000000000041

[32] Fischgrund JS , Mackay M , Herkowitz HN , Brower R , Montgomery DM , Kurz LT . 1997 Volvo Award winner in clinical studies. Degenerative lumbar spondylolisthesis with spinal stenosis: a prospective, randomized study comparing decompressive laminectomy and arthrodesis with and without spinal instrumentation. Spine, 1997, 22: 2807–2812. 9431616 10.1097/00007632-199712150-00003

[33] Huang W , Han Z , Liu J , Yu L , Yu X . Risk factors for recurrent lumbar disc herniation: a systematic review and meta‐analysis. Medicine, 2016, 95: e2378. 26765413 10.1097/MD.0000000000002378 PMC4718239

[34] Buchmann N , Preuß A , Gempt J , *et al* Outcome after surgical treatment for late recurrent lumbar disc herniations in standard open microsurgery. World Neurosurg, 2016, 89: 382–386. 26882970 10.1016/j.wneu.2016.02.028

